# Can slide positivity rates predict malaria transmission?

**DOI:** 10.1186/1475-2875-11-117

**Published:** 2012-04-18

**Authors:** Yan Bi, Wenbiao Hu, Huaxin Liu, Yujiang Xiao, Yuming Guo, Shimei Chen, Laifa Zhao, Shilu Tong

**Affiliations:** 1 School of Public Health and Social Work, Institute of Health and Biomedical Innovation, Queensland University of Technology, Victoria Park Road, Kelvin Grove, 4059, Brisbane, Australia; 2 Yunnan Center for Disease Control and Prevention, 158 Dongsi Road, 650022, Kunming, China; 3 School of Population Health, University of Queensland, Herston Road, Herston, 4006, Brisbane, Australia; 4 Mengla Center for Disease Control and Prevention, Mengla Nan Road, 666300, Xishuangbanna, China

**Keywords:** Malaria transmission, Slide positivity rates, Malaria elimination, International border areas, China

## Abstract

**Background:**

Malaria is a significant threat to population health in the border areas of Yunnan Province, China. How to accurately measure malaria transmission is an important issue. This study aimed to examine the role of slide positivity rates (SPR) in malaria transmission in Mengla County, Yunnan Province, China.

**Methods:**

Data on annual malaria cases, SPR and socio-economic factors for the period of 1993 to 2008 were obtained from the Center for Disease Control and Prevention (CDC) and the Bureau of Statistics, Mengla, China. Multiple linear regression models were conducted to evaluate the relationship between socio-ecologic factors and malaria incidence.

**Results:**

The results show that SPR was significantly positively associated with the malaria incidence rates. The SPR (β = 1.244, p = 0.000) alone and combination (SPR, β = 1.326, p < 0.001) with other predictors can explain about 85% and 95% of variation in malaria transmission, respectively. Every 1% increase in SPR corresponded to an increase of 1.76/100,000 in malaria incidence rates.

**Conclusion:**

SPR is a strong predictor of malaria transmission, and can be used to improve the planning and implementation of malaria elimination programmes in Mengla and other similar locations. SPR might also be a useful indicator of malaria early warning systems in China.

## Background

Malaria is one of the major public health problems in China, especially in Yunnan Province, which has significant mortality, morbidity and economic burden. Yunnan Province is a malarial hyper-endemic area and had the highest number of malaria cases and deaths for more than 10 years until 2005 in China [1,2]. The outbreaks of malaria happen annually along border areas in Yunnan, China. The likelihood of imported malaria cases has been increased along the border areas between Yunnan and Myanmar, Laos and Vietnam over recent years, due to increased trade and tourism in these areas [2,3]. In order to control malaria it is important to enhance disease surveillance and evaluation of malaria transmission [4,5] in this endemic region.

The intensity of malaria transmission can be estimated using different indicators such as annual blood examination rate (ABER), annual parasite index (API), slide positivity rate (SPR) and the incidence of malaria [6-10]. In China, the annual malaria incidence is commonly used. Malaria incidence includes numbers of laboratory-confirmed malaria cases and other cases diagnosed with clinic symptoms (e.g. fever) as a numerator and the local population as a denominator. The local population size might be under- or overestimate because census is only carried out once 10 years in China. Huge population movement is common due to economic development in China in the last three decades. Thus, malaria incidence might be inaccurate due to limited health care resources [7] or under- or overestimates of population size [11]. It is important to estimate the burden of malaria accurately for planning public health interventions. Slide positivity rate (SPR) has been used as a surrogate to measure the incidence of malaria [7,9,12,13], to define the level of malaria endemicity [11], and to identify malaria high risk areas [14]. This is a principal monitoring indicator in the malaria elimination programme in China for the period 2010 and 2020 and it has been monitored since the 1980s [15,16] through the malaria annual reporting system. The changes in malaria incidence can be estimated from the SPR trends [7]. Some studies have demonstrated that SPR has steadily decreased with the decline in malaria incidence [8,12], while others found that the annual parasite index (API) increased, but SPR kept steady at the same level over 20 years [8].

The development of the malaria early warning system (MEWS) has been started based on the surveillance system in China over recent years [17,18]. However, these studies are limited to climatic indicators and did not take advantage of monitoring indicators, which can help improve malaria prevention and control, especially in the early stage of malaria elimination. Moreover, the relationship between SPR and the incidence of malaria is not clear in the border areas of Yunnan Province, China. This study aimed to examine the role of SPR in monitoring malaria transmission, and improve the planning and implementation of malaria control and prevention programmes.

## Methods

### Study site

**Figure 1:**
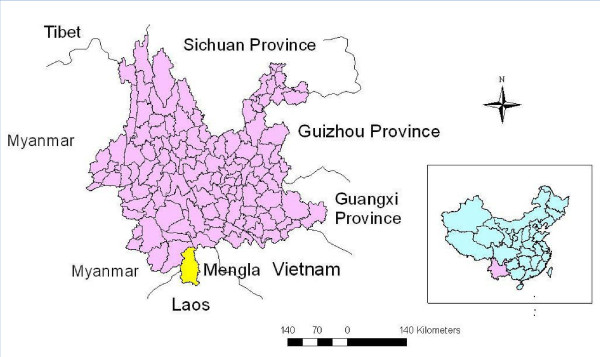
The location of Mengla County, Yunnan Province, China.

Mengla County is in south Yunnan Province and ranges from 21° 09' to 22° 24'N, 101° 05' to 101° 50'E, bordering Myanmar to the west and Laos to the east, south and south-west as well as other counties of Yunnan Province to the north (Figure 1). It has an area of 7,093 sq km with an international border of 740.8 km. Mengla County includes 10 townships and four farms with a population of 0.2 million. Its elevation ranges from 480 m to 2,023 m. It is a high malaria transmission region. Malaria has been the top infectious disease for decades and was ranked the first (accounted for 46.7% of total cases) in all infectious diseases in 2003. Mengla was ranked top six for its annual malaria incidence (400.4/100,000) among the 2,353 counties of China during 1994–1998 [19]. Malaria becomes one of the major public health problems in this region. Increased travel across international border (China-Myanmar and China-Laos) aggravates the burden of malaria [2,20]. In the national malaria elimination programme of China launched in 2010, Mengla was identified as one of the 75 first line counties in China and will achieve the goal of no indigenous malaria cases by 2017 and malaria elimination by 2020 [15].

### Data collection

Data on annual malaria cases and SPR in all fever patients were obtained between 1993 and 2008 from the malaria annual reporting system in the Mengla Center for Disease Control and Prevention (CDC), China. Mengla is one of the sentinel counties selected for both national and provincial malaria surveillance, and has kept good records for malaria. The dominant species of parasitized by malaria is *Plasmodium vivax*, but *Plasmodium falciparum* infections also exist in this county. The ratio of *P. vivax* to *P. falciparum* cases was 4:1 [21]. Both species were combined in this study. SPR defined as the number of laboratory-confirmed positive slides examined per 100 slides, expressed as a percentage [7,10]. The calculation of SPR is

(1) $$\text{SPR\textbackslash{};for\textbackslash{};a\textbackslash{};year}=\left(\text{number\textbackslash{},of\textbackslash{},positive\textbackslash{},slides}/\text{total\textbackslash{},slides\textbackslash{},examined}\right)\times 100$$

Blood smears of febrile patients were examined, and confirmed by microscope and/or by rapid diagnostic test.

Data on climatic variables (including annual average relative humidity, mean maximum temperature (Tmax), mean minimum temperature (Tmin) and rainfall); and the annual average income per capita of farmers and the population size of this county for the same period were retrieved from the Mengla Bureau of Meteorology and the Mengla Bureau of Statistics, respectively. An ethical approval was granted by the Human Research Ethics Committee, Queensland University of Technology (#1000000573).

### Data analysis

Spearman’s correlation analyses were conducted to evaluate the correlations between SPR and the incidence of malaria, as well as other independent variables. Six step-wise multiple linear regression models were employed to examine the effects of SPR on malaria transmission after adjusting for confounding variables. Square root transformation was applied to the malaria incidence to assure the normality to satisfy the assumption of linear regression analysis. The Durbin-Watson (DW) statistic was used to detect the presence of autocorrelation (a relationship between values separated from each other) in the residuals (prediction errors) from the above regression analysis. If the DW statistic is substantially equal to two, it indicates no autocorrelation. Akaike Information Criterion (AIC) was used to select the most suitable model. All data analyses were conducted using SPSS for WinWrap Basic (PASW Statistics, Version 18).

## Results

**Figure 2:**
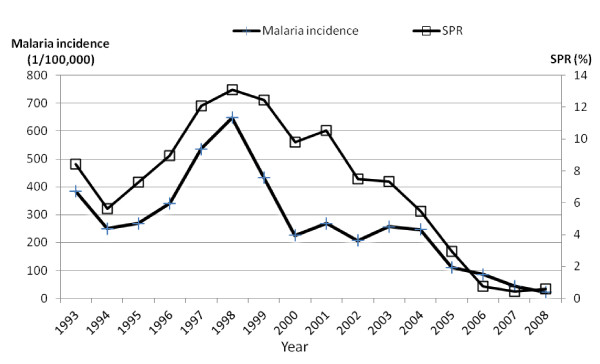
Malaria incidence and slide positivity rates (SPR) in Mengla County, China, 1993–2008.

**Figure 3:**
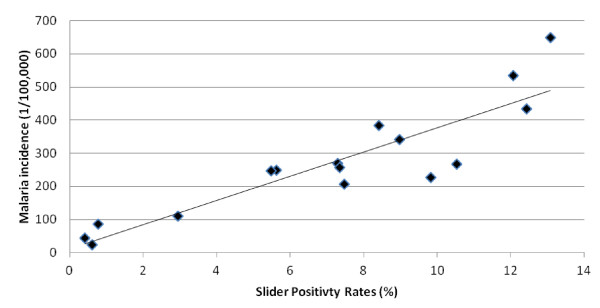
The relationship between slide positivity rates and crude malaria incidence in Mengla.

Figure 2 shows the annual pattern of malaria incidence and SPR in Mengla County. In this hyper-endemic region, a total of 8,962 malaria cases were reported and annual malaria incidence rates ranged from 23 to 648 per 100,000, while the SPR varied between 0.42% and 13.08% from 1993 to 2008. The scatter plot with regression line depicts the crude relationships between incidence rates of malaria and SPR (Figure 3). The plot reveals that incidence rates of malaria were positively associated with SPR.

Spearman correlations between malaria incidence and socio-environment variables show (Table 1) that SPR (r = 0.85, p < 0.01), income (r = −0.76, p < 0.01) and humidity (r = 0.57, p < 0.05) were statistically significantly associated with malaria incidence. However, there was no significant association between other climatic variables and annual malaria incidence.

**Table 1:** Spearman correlations between malaria incidence and social and climatic variables, 1993-2008

Variables	SPR	Income	Tmax	Tmin	Rainfall	Humidity
Income	-.544^*^					
Tmax	−0.38	0.26				
Tmin	−0.04	0.39	0.27			
Rainfall	0.15	−0.35	−0.45	−0.41		
Humidity	.792^**^	-.567^*^	-.515^*^	−0.22	0.39	
Malaria incidence	.853^**^	-.756^**^	−0.23	−0.20	0.10	.568^*^
* p < 0.05	**p < 0.01					

*SPR* Slide positivity rates, *Tmax* Maximum temperature, *Tmin* Minimum temperature.

Six models have been used to evaluate the association between malaria incidence and predictors (Table 2). Model 1 shows SPR (β = 1.244, p = 0.000) alone can explain as high as 85% of the variation in the response variable. This provides strong evidence that SPR is a very good surrogate measure for the malaria incidence rates. Models 2–6 show that the inclusion of the additional covariates of Tmax, income and humidity moderately improved the model fit with the increase of adjusted R^2^ (88-95%) and DW value (0.57-2.11), and decrease of AIC (75.93-65.62) in these models. Model 6 (R^2^: 95%, AIC: 65.62) is chosen as the optimal model due to its best goodness-of-fit of the data. In summary, the best fitting model includes SPR, income, maximum temperature and humidity as the predicting variables for the annual malaria incidence.

**Table 2:** Association between malaria incidence and SPR in Mengla, China 1993-2008

**Models**		**SPR**		**Adjusted R**^**2**^	**AIC**	**D-W (p-value)**
	β	**S.E.**	**P**			
Model 1	1.244	0.133	0.000	0.851	75.93	0.78 (=0.001)
Model 2	1.359	0.128	0.000	0.884	74.36	0.57(<0.001)
Model 3	1.039	0.137	0.000	0.895	72.79	1.12(=0.005)
Model 4	1.152	0.110	0.000	0.939	67.32	1.21(=0.007)
Model 5	1.318	0.163	0.000	0.924	70.67	1.54(=0.045)
Model 6	1.326	0.131	0.000	0.951	65.62	2.11(=0.246)

*SPR* Slide positivity rates, *D-W* Durbin-Watson, *AIC* Akaike Information Criterion, Model 1: SPR, Model 2: SPR + Tmax, Model 3: SPR + income, Model 4: SPR + Tmax + income, Model 5: SPR + Tmax + humidity, Model 6: SPR + Tmax + income + humidity;

Table 3 displays crude and adjusted results from linear regression analyses. In crude models, four predictors were tested individually. Their adjusted R^2^ were −3.2% (Tmax), 45% (humidity), 47% (income) and 85% (SPR), respectively. In the multi-variable models, without SPR, only 54% of variation of malaria incidence was accounted for by the other three independent predictors (Tmax, humidity and income), whereas 95% of variation of the malaria incidence was explained after SPR was added to the model.

**Table 3:** Regression coefficients of the best model

		**Crude**			**Without SPR**		**Adjusted**		**With SPR**	
**Variables**	β	**S.E.**	**P**	**Adjusted R**^**2**^	β	**S.E.**	**P**	β	**S.E.**	**P**
Tmax	−2.57	3.522	0.479	−0.032	2.25	2.717	0.424	2.43	0.883	0.019
Humidity	2.15	0.589	0.003	0.450	1.56	0.777	0.068	−0.68	0.335	0.069
Income	−0.01	0.002	0.002	0.469	0.004	0.002	0.081	−0.003	0.001	0.001
SPR	1.24	0.133	0.000	0.851				1.326	0.131	0.000
Adjusted R^2^					0.536			0.951M		

Multiple linear regression model: SPR + Tmax + humidity + income (model 6).

Table 3 also shows that SPR (β = 1.326, p < 0.001) is a significantly independent predictor of malaria incidence after adjustment for Tmax, humidity and income. Keeping other independent variables constant, every 1% increase in SPR corresponds to an increase of 1.76/100,000 (the squared malaria incidence) in malaria incidence rates.

**Figure 4:**
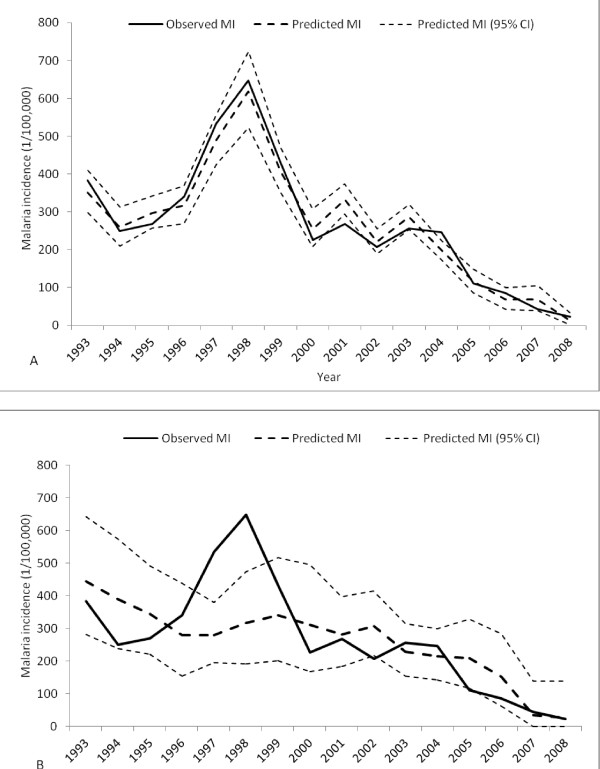
Regressive forecasts of annual malaria incidence in Mengla, China, 1993–2008, including SPR (A); and not including SPR (B).

Figure 4 shows the results of the regressive forecast chart in which Figure 4-A included SPR and Figure 4-B did not. Figure 4-A indicates that the predicted and the observed value of annual squared root malaria incidence rates matched well. The incidence rates in 1999, and in 2003, were theoretically predicted by the model and validated by the observed values. However, for Figure 4-B the predicted and the observed value of the malaria incidence cannot be matched well, especially in year 1997–98. The observed values of these two years are out of the confidence interval. There is a wider confidence interval in Figure 4-B than in 4-A. All results stated that the regressive forecast of annual malaria incidence with SPR is more accurate than that without SPR in Mengla County over the study period.

## Discussion

The results of this study indicate that SPR is a strong predictor of malaria incidence. SPR varied between 5.48% and 13.98% from 1993 to 2004 in Mengla. SPR under 2.9% is considered the absence of indigenous transmission [9]. Evidently, there is indigenous malaria transmission in Mengla [2,20]. Less than 5% of SPR is considered the transition from the control stage to the pre-elimination stage [22] which implied that Mengla went through pre-elimination malaria after 2004. Five *Anophiline* species have been identified to be vectors of malaria in Yunnan Province [2,23]. *Anopheles minimus* is the major vector in this endemic border area - Mengla [2,20].

SPR has been used as a surrogate of malaria incidence [7,9,12]. In Ugandan, SPR provided a useful measure to estimate malaria incidence among children [7]. To measure malaria transmission at a pre-elimination stage, SPR was used as an indicator to evaluate a malaria control programme on the island of Principe [12]. In current study, the decrease in SPR corresponded to the malaria incidence decline. This result is consistent with the result of other studies in which changes of SPR provided an alternative method for estimating changes in the incidence of malaria [7,12]. A downward trend in SPR in Mengla is in accordance with the decline of both *P.vivax* and *P. falciparum* malaria incidence in Yunnan [24]. After 2005, both SPR and malaria incidence sharply decreased in Mengla. This may be due to the impact of the Mekong Roll Back Malaria program (2002–2004) and the Global Fund (Round one) between 2003 and 2008, especially with the free treatment for malaria infection financed by the Global Fund in Mengla County since 2005.

Malaria transmission is greatly affected by socioeconomic conditions [25,26]. Low-middle income was significantly associated with malaria transmission in Indonesia [27]. The disappearance of malaria in some areas of Europe was associated with economic development [28]. In this study, income was negatively associated with malaria incidence. The decrease in malaria incidence was consistent with the increase in income. This can be explained by the development of the general economy in Mengla County in the last two decades. Mengla is a poor region. Twenty-six ethnic minority groups accounted for 72% of the total population, and approximately 96% of Mengla is mountainous. The main income is from rice, rubber, cane sugar and tea (it is the place of origin of Pu Er tea) [29]. The local economy has been improved since the 1990s by planting rubber trees, tropical fruit trees, tea, and an increase in trade with Laos, Myanmar and other Mekong-river region countries. The incomes of farmers have gradually increased, which has led to better living conditions and improvements in sanitation and health. These improved socio-economic conditions may be one of the key reasons for the decreased malaria pattern in this region. Further investigation of the association between socioeconomic conditions and malaria transmission is warranted in this endemic area.

In this study, relative humidity has a significant positive association with malaria incidence. Relative humidity appears to have an effect on malaria transmission indirectly, as humidity may affect the development of the parasite, and the activity and survival of anopheline mosquitoes [27]. More humid and hotter than usual conditions may increase anopheline survival, thus resulting in an increase in outbreaks of malaria [30]. However, low humidity could reduce the numbers of the mature mosquitoes [31], therefore resulting in no malaria transmission [27]. As a tropical rain forest area situated just south of the Tropic of Cancer, Mengla has wet and hot weather, which provides mosquitoes with favourable breeding sites.

Temperature has an important effect on the transmission cycle of the malaria parasite and mosquito survival [25,32]. Temperature is considered to play a crucial role in malaria transmission, which was identified by other studies [24,33-36] and is reported to be a predictor of malaria transmission. In a previous study in 2008, a positive association between minimum temperature, maximum temperature and malaria incidence based on monthly time series data was found in Mengla County [21]. However, the association between temperature and malaria incidence was not observed in current study. This may because annual weather variables are used for analysis. In multiple linear regression analysis, however, maximum temperature became a significant predictor of malaria transmission after adjustment for other factors. Maximum temperature and another three predictor factors together explained 95% of variance of malaria incidence.

Malaria transmission is influenced by various factors including climatic [37,38] and non-climatic factors [39-41]. The spatial and seasonal distribution of malaria is largely determined by climate [42], and climatic factors (e.g. rainfall, temperature and humidity) have been widely used and recognized in the MEWS [43,44]. However, climatic factors are not enough for MEWS, which requires comprehensive and integrated indicators. To predict the timing and severity of malaria epidemics in MEWS, epidemiological surveillance indicators (for example SPR) should be considered [45,46]. Blood examination of parasite appearance is a key indicator in the early detection of malaria transmission and it is compulsorily reported in China. The use of SPR can obviously assist the development of MEWS in malaria elimination program in China. It can also be used to evaluate the malaria surveillance systems in China.

This study has several strengths. Firstly, this is the first study to examine the role of SPR in monitoring malaria transmission at a county level in China. Secondly, the model developed fitted the data quite well. The SPR alone, and combination with other predictors can explain 85% and 95% of variation in malaria transmission, respectively. Finally, the results of this study may help plan and implement malaria control and prevention interventions in the field.

The limitations of this study should also be acknowledged. Firstly, SPR and income were collected annually, and we were unable to examine the seasonal pattern of malaria transmission and conduct any finer analyses (e.g. monthly). Secondly, some other factors (e.g. mosquito density, movement of the people across the border and vegetation coverage) may play a role in the transmission of malaria. Because of the lack of the data, these factors were not adjusted for in the model. Finally, the data for this study were only collected from Mengla, Yunnan Province. There was no detailed information on *P. vivax* and *P. falciparum,* and the recurrence of *P. vivax* infection was also not considered, hence they could not be analysed separately. Thus, caution is needed when the findings of this study are generalized to other locations.

In conclusion, SPR was significantly associated with malaria incidence and identified as a strong predictor of malaria transmission in Mengla County. The results of this study support the use of SPR. The multi-variable regression model developed in this study may have implications for the global malaria elimination campaign. The improved understanding of the relationship between SPR and malaria transmission will assist in the establishment of a malaria early warning system to predict this wide spread disease in endemic areas.

## Competing interests

The authors declare that they have no competing interests.

## Authors’ contributions

WBH and SLT initiated the study. YB, WBH and YMG designed the study and directed its implementation, including data analysis and interpreting. HXL, YJX, SMC, LFZ and YB performed field data collection. SLT supervised the study and YB drafted the manuscript. All authors contributed to the manuscript edit, review and revising, and approved the final version of the manuscript.
